# Zi Shen Wan Fang Attenuates Neuroinflammation and Cognitive Function *Via* Remodeling the Gut Microbiota in Diabetes-Induced Cognitive Impairment Mice

**DOI:** 10.3389/fphar.2022.898360

**Published:** 2022-07-15

**Authors:** Jiangwei Shi, Qingsheng Yin, Lin Zhang, Yu Wu, Pengrong Yi, Mengqing Guo, Huhu Li, Liuyi Yuan, Zixuan Wang, Pengwei Zhuang, Yanjun Zhang

**Affiliations:** ^1^ Department of Integrated Rehabilitation, First Teaching Hospital of Tianjin University of Traditional Chinese Medicine, Tianjin, China; ^2^ National Clinical Research Center for Chinese Medicine Acupuncture and Moxibustion, Tianjin, China; ^3^ Chinese Materia Medica College, Tianjin University of Traditional Chinese Medicine, Tianjin, China; ^4^ School of Integrative Medicine, Tianjin University of Traditional Chinese Medicine, Tianjin, China; ^5^ State Key Laboratory of Component-based Chinese Medicine, Tianjin University of Traditional Chinese Medicine, Tianjin, China; ^6^ Haihe Laboratory of Modern Chinese Medicine, Tianjin University of Traditional Chinese Medicine, Tianjin, China

**Keywords:** ZSWF, DCI, inflammation, intestinal barrier, gut microbiome

## Abstract

**Background** **:** Cognitive dysfunction is a critical complication of diabetes mellitus, and there are still no clinically approved drugs. Zi Shen Wan Fang (ZSWF) is an optimized prescription composed of Anemarrhenae Rhizoma, Phellodendri Chinensis Cortex, and Cistanches Herba. The purpose of this study is to investigate the effect of ZSWF on DCI and explore its mechanism from the perspective of maintaining intestinal microbial homeostasis in order to find an effective prescription for treating DCI.

**Methods:** The diabetes model was established by a high-fat diet combined with intraperitoneal injections of streptozotocin (STZ, 120 mg/kg) and the DCI model was screened by Morris water maze (MWM) after 8 weeks of continuous hyperglycemic stimulation. The DCI mice were randomly divided into the model group (DCI), the low- and high-ZSWF–dose groups (9.63 g/kg, 18.72 g/kg), the mixed antibiotic group (ABs), and the ZSWF combined with mixed antibiotic group (ZSWF + ABs). ZSWF was administered orally once a day for 8 weeks. Then, cognitive function was assessed using MWM, neuroinflammation and systemic inflammation were analyzed by enzyme-linked immunosorbent assay kits, intestinal barrier integrity was assessed by hematoxylin-eosin (HE) staining and Western blot and high performance liquid chromatography tandem mass spectrometry (UPLC-MS/MS). Furthermore, the alteration to intestinal flora was monitored by 16S rDNA sequencing.

**Results:** ZSWF restored cognitive function in DCI mice and reduced levels of proinflammatory cytokines such as IL-1β, IL-6, and TNF-α. Moreover, ZSWF protected the integrity of the intestinal barrier by increasing intestinal ZO-1 and occludin protein expression and decreasing urinary lactulose to mannitol ratio. In addition, ZSWF reshaped the imbalanced gut microbiota in DCI mice by reversing the abundance changes of a wide range of intestinal bacteria at the phyla and genus levels*.* In contrast, removing gut microbiota with antibiotics partially eliminated the effects of ZSWF on improving cognitive function and reducing inflammation, confirming the essential role of gut microbiota in the improvement of DCI by ZSWF.

**Conclusion:** ZSWF can reverse cognitive impairment in DCI mice by remolding the structure of destructed gut microbiota community, which is a potential Chinese medicine prescription for DCI treatment.

## Introduction

Diabetes mellitus (DM) is a common metabolic disease with increasing incidence. Cumulative studies confirm that DM is a key risk factor for cognitive impairment (Zilliox et al., 2016; Biessels and Despa, 2018; Biessels and Whitmer, 2020). Although many excellent studies of DCI exist, its potential mechanisms remain elusive and effective drugs are lacking. Several studies suggested that DCI might be attributed to cerebrovascular dysfunction (Prakash et al., 2012), neuroinflammation (Rom et al., 2019), and metabolic disorders (Zhang et al., 2020a). In recent years, emerging studies noticed that intestinal microbiota structure and function disorders are closely related to brain function. Gut-brain crosstalk is a complex network system, which maintains the stability of gastrointestinal tract on the one hand and affects the brain homeostasis on the other hand (Rhee et al., 2009). Intestinal microbes regulate brain homeostasis through multiple pathways, among which intestinal barrier-inflammation is one of the main pathways (Rogers et al., 2016). Next, the intestinal barrier plays a crucial role in maintaining of intestinal microbiome and peripheral homeostasis. In contrast, intestinal microbiome dysbiosis can provoke disruption of the intestinal barrier. Increased intestinal permeability can cause excessive translocation of bacterial lipopolysaccharide into the bloodstream, which can trigger systemic inflammation (Zhang et al., 2019). Microbiota-derived inflammatory response ultimately leads to neuroinflammation and neuronal damage (Bairamian et al., 2022).

Increasing evidence indicates that the structural changes of intestinal microbiota are associated with the development of DCI (Xu et al., 2017). Clinical studies found significant differences in gut microbiome composition between diabetic patients with and without cognitive impairment (Zhang et al., 2021a). Also, preclinical studies suggested that DCI was associated with alterations of the gut microbiome (Gao et al., 2019). Furthermore, Yu et al. (2019) transplanted fecal bacteria from DCI and non-DCI mice into the gut of pseudo-germ-free mice, respectively, and they found that the escape latency was significantly longer in pseudo-germ-free mice receiving DCI mouse fecal bacteria than those receiving non-DCI mouse fecal bacteria. These studies suggest that maintaining intestinal microbial homeostasis is an effective strategy for the prevention and treatment of DCI.

Traditional Chinese medicine (TCM) believes that “poison damage brain collateral” and “deficiency of kidney essence” are the main pathogenesis of DCI, and “clearing heat-fire and detoxifying” and “invigorating kidney for protecting semen” are the key therapeutic principles. Anemarrhenae Rhizoma and Phellodendri Chinensis Cortex are the most commonly used heat-clearing drug pair in clinical practice, and they were originally derived from “Tong Guan Wan.” Previous modern pharmacological evidence suggests that Anemarrhenae Rhizoma and Phellodendri Chinensis Cortex drug pair exhibit an effect on improving diabetes-related complications (Zhang et al., 2014), and their active ingredients demonstrate a clear protective effect on cognitive function (Liu et al., 2012; Xian et al., 2013; Liang et al., 2017; Piwowar et al., 2020). However, studies also found that the bioavailability of saponins and alkaloids (the main components of Anemarrhenae Rhizoma and Phellodendri Chinensis Cortex, respectively) is low (Singh and Chaudhuri, 2018; Baldim et al., 2020), suggesting that regulation of intestinal microbial homeostasis may be the key pathway for them to improve diabetes-related complications. Furthermore, considering that the treatment of diabetes-related complications is a long process and that Anemarrhenae Rhizoma and Phellodendri Chinensis Cortex are severe cold medicines, the compatibility of warm drugs to neutralize cold is the basic principle of the compatibility of TCM prescriptions. Cistanches Herba (*Cistanche deserticola* Y. C. Ma), a classic Chinese medicine with the effect of tonifying kidney and nourishing essence, is warm in property. Also, modern pharmacological studies suggest that Cistanches Herba demonstrates the function of maintaining intestinal flora homeostasis and neuroprotection (Li et al., 2017; Wang et al., 2017). Therefore, under the guidance of basic theories of TCM, we formed ZSWF by the compatibility of the Cistanches Herba (*C. deserticola* Y. C. Ma) and Anemarrhenae Rhizoma (*Anemarrhena asphodeloides* Bge)-Phellodendri Chinensis Cortex (*Phellodendron chinense* Schneid) herb pair, hoping to find a new prescription to treat DCI.

In this study, we investigated the effects of ZSWF on the cognitive function of DCI mice, and we explored the possible mechanism by regulating intestinal flora. Cognitive function, neuroinflammation and systemic inflammation, intestinal barrier integrity, and gut microbiome diversity were examined. Furthermore, an antibiotic intervention was used to assess the possibility of a causal relation between ZSWF improving cognitive and gut microbiota.

## Methods

### Preparation of Zi Shen Wan Fang Extract

ZSWF is composed of Anemarrhenae Rhizoma (dried rhizome of *A. asphodeloides* Bge.), Phellodendri Chinensis Cortex (dried bark of *P. chinense* Schneid.), and Cistanches Herba (dried fleshy stem of *C. deserticola* Y. C.Ma) with a ratio of 1:1:1 in weight. The drug materials of Anemarrhenae Rhizoma and Phellodendri Chinensis Cortex were purchased from Hebei anguo Chinese Herbal Medicine Co., Ltd., and Cistanches Herba was purchased from Inner Mongolia Mandera Biological Technology Co., Ltd. These herbs were identified by professor Tianxiang Li (TianJin University of Traditional Chinese Medicine) and were extracted according to our previous method (Zheng et al., 2018). In simple terms, Anemarrhenae Rhizoma and Phellodendri Chinensis Cortex were mixed at the ratio of 1:1 by weight, then extracted by reflux for 3 times with 80% ethanol of 8 times (2 h each time), and filtrate was collected. The same weight Cistanches Herba was first extracted by reflux for 3 times with 8 times the amount of 80% ethanol (2 h each time), and filtrate was collected and then extracted with 10 times the amount of distilled water for 3 times (2 h each time). Next, the volume of liquid required for ingastric administration of mice during the treatment cycle was calculated, and all filtrate was mixed and concentrated to 0.94 g/ml ZSWF crude extract by rotary evaporator, which was separated and stored in a refrigerator at −80°C for use. Moreover, our previous study systematically investigated the main chemical components of ZSWF extract (Zheng et al., 2018), including *in vitro* components (e.g., Neomangiferin, Berberine, TimosaponinBⅡ, and Cistansinenside A), absorbed components (e.g., Mangiferin, Timosaponin C, and 3/5-O-Feruloylquinic acid), as well as prototypical (e.g., Phellodenrine, Tetrahydropalmatine, and Oxoberberine) and metabolic components [e.g., 3,4-dihydroxybenzenepropionic acid and (20R, 25S)-timosaponin AI] in feces.

### Establish the Diabetic Cognitive Impairment Model and Administration Method

Two hundred male C57BL/6J mice (8 weeks-old) were purchased from Beijing Vital River Laboratory Animal Technology Co., Ltd., [SCXK (Jing) 2016-006]. The animals were housed in the Laboratory Animal Center of Tianjin University of Traditional Chinese Medicine under a standard laboratory condition (temperature 22 ± 2°C, humidity at 50 ± 15%, 12-h-light/12-h-dark cycle) and were given *ad libitum* access to water and food. All experimental procedures were approved by the Animal Ethics Committee of Tianjin University of Traditional Chinese Medicine (TCM-LAEC2019083, Tianjin, China).

After 1 week of adaptive feeding, the diabetic mouse model was replicated as previously reported (Kusakabe et al., 2009). In simple terms, mice were intraperitoneally injected with 120 mg/kg streptozotocin (STZ, Sigma, United States) after being fed a high-fat diet with 60% energy from fat for 3 weeks (Beijing Vital River Laboratory Animal Technology Co., Ltd.). One week after injection, fasting blood glucose (FBG) of mice after 12 h of fasting was detected, and mice with FBG > 11.1 mmol/L were selected to continue high-fat diet feeding. After 8 weeks of continuous hyperglycemic stimulation, mice with cognitive impairment were screened with MWM (detailed in 2.3) and used in follow-up studies.

To investigate the effect of ZSWF on the cognitive function of DCI mice and its potential mechanism, the mice with cognitive impairment were randomly divided into five groups: vehicle treated group (DCI), ZSWF low dose (9.36 g/kg, clinical equivalent dose) treated group (ZSWFL), ZSWF high dose (18.72 g/kg, 2 times the clinical equivalent dose) treated group (ZSWFH), mixed antibiotic (natamycin 3 mg/ml, neomycin 2 mg/ml and bacitracin 2 mg/ml) treated group (ABs), and ZSWF combined with mixed antibiotic treated group (ZSWF + ABs). Mice in the treatment group were orally gavaged with crude extract of ZSWF for 8 weeks, and mice in Con group and DCI group were gavaged with equal volume of distilled water. The control group was fed the normal diet, and the other groups were kept on HFD during drug administration. Weight was measured every 2 weeks, and FBG was measured weekly during continuous treatment.

### Morris Water Maze Experimental Evaluation of Cognitive Function

To screen mice with abnormal cognitive function after 8 weeks of hyperglycemia and investigate the effect of ZSWF treatment on cognitive function, the MWM experiment was performed as previously described (Morris., 1984). In short, to acclimate to the maze environment, all the mice were allowed to swim freely in the maze without a platform for 1 min the day before the experiment began. Next, the positioning navigation experiment was conducted for five consecutive days, during which the mice were allowed to swim freely for 60 s. If they did not reach the platform within 60 s, the mice were slowly guided to the platform and held there for 10 s. In the space exploration experiment, the mice were allowed to swim freely for 60 s in a maze where the platform was removed. During this process, the time to first reach the original platform, the duration in the platform quadrant, and the frequency of crossing the platform were recorded. During the whole test, data acquisition is completed by the automatic image surveillance and processing system.

### Cresyl Violet Staining

To investigate the effect of ZSWF on hippocampal neurons of DCI mice, cresyl violet staining was performed. At the end of the treatment, the mice were deeply anesthetized by inhalation with 4% isoflurane, and blood samples were collected. Apical perfusion with PBS was then performed to remove residual blood from the brain tissue, and whole brain tissue was collected. After fixation with 4% paraformaldehyde, the brain tissue was dehydrated with gradient sucrose solution (10%, 20%, and 30%, respectively), and the coronal sections with thickness of 10 μm were obtained using a frozen slicer. Sections were stained with 0.1% cresol violet solution, dehydrated with ascending grades of alcohol, cleared with xylene, mounted with neutral resin, and images were taken using Leica DM4000B biological microscope (Beijing, China).

### Enzyme-Linked Immunosorbent Assay

To investigate the effects of ZSWF on neuroinflammation in DCI mice, the mice were sacrificed by cervical dislocation after deep anesthesia. Then, the skull was cut along the midline of the skull to expose the whole brain tissue, the cortex was separated to expose the hippocampus, and the hippocampus was collected with bamboo sticks and stored at −80°C for detection. Also, commercially available enzyme-linked immunosorbent assay (ELISA) kits were used to measure the hippocampus IL-6 (JYM0012Mo, Colorful-Gene, 2–150 pg/ml), IL-1β (JYM0531Mo, Colorful-Gene, 1.5–100 pg/ml), TNF-α (JYM0218Mo, Colorful-Gene, 8–500 pg/ml), and monocyte chemotaxis protein-1 (MCP-1, JYM0099Mo, Colorful-Gene, 6–450 pg/m) according to manufacturer’s instructions. To investigate the effects of ZSWF on proinflammatory cytokines in peripheral circulation in DCI mice, blood samples from each group were collected and centrifuged at 3,000 g for 15 min. The levels of IL-6, IL-1β, TNF-α, and INF-γ (abs552811, Absin Bioscience Inc., 7.81–500 pg/ml) in the serum were also detected using ELISA kits. Furthermore, to investigate the effect of ZSWF on the integrity of intestinal mucosal barrier in DCI mice, colon tissue approximately 5 cm connected to the cecum of mice in each group was taken, colon contents were scraped, and then, the colon and colon contents were stored in EP tubes, respectively. Next, colon tissue was lysed with lysate, and the content of mucin-2 (MUC2) in colon was determined by the ELISA kit (SEA705Mu, Cloud Clone Biotechnology Co., LTD., 0.78–50 ng/ml). The colon contents were diluted with 0.9% Nacl, homogenized and centrifuged (3,000 g, 15 min), and the content of secretory immunoglobulin A (SlgA) in the colon contents was determined by the ELISA kit (SEA641Mu, Cloud Clone Biotechnology Co., LTD., 0.156–10 ng/ml). All experimental procedures were performed according to the manufacturer’s instructions. The optical density at 450 nm was obtained with an ELISA microplate reader (Infinite^®^ 200 PRO, Tecan, Swit). There were six biological repetitions in each group for proinflammatory cytokines and five biological repetitions in each group for Muc2 and SlgA.

### Hematoxylin-Eosin Staining of Colon

To evaluate the effect of ZSWF on intestinal barrier integrity in DCI mice, the colon was fixed in 4% paraformaldehyde for 24 h, and then, the paraffin section was performed. Sections with a thickness of 0.5 μm were stained with hematoxylin (5 min) and 0.5% eosin (3 min), and the histopathological images of the colon were taken with a Leica DM4000B biologic microscope.

### Western Blot Analysis

To investigate the effect of ZSWF on intestinal permeability of DCI mice, Western blot analysis was used to detect the protein expressions of occludin and ZO-1. Proteins from colon tissues were extracted and quantified using the BCA protein quantification kit. Total proteins were separated by SDS polyacrylamide gel electrophoresis (SDS-PAGE) and transferred onto a polyvinylidene fluoride (PVDF) membrane by wet transfer apparatus. Tight junction protein associated antibodies ZO-1 (ab216880, Abcam, United Kingdom) and occludin (ab216327, Abcam, United Kingdom) were used to incubate the bands overnight at 4°C, where β-actin (13E5, CST, United States) was used as a reference protein, and then, goat anti-rabbit secondary antibodies (ab205718, Abcam, United Kingdom) were used to incubate the bands at room temperature for 2 h. In conclusion, the immunoreactive bands were visualized using enhanced chemiluminescence reagents, and the luminescence intensity was quantitatively analyzed using Image-pro Plus6.0.

### HPLC-MS/MS Analysis Lactulose/Mannitol Ratio in Urine

To observe the effect of ZSWF on intestinal barrier integrity in DCI mice, the ratio of lactulose to mannitol in urine was determined by HPLC-MS/MS according to the previous method (Kubica et al., 2012). At the end of treatment, mice were given a 2:1 lactulose-mannitol solution, and urine was collected in a metabolic cage for 6 h. The supernatant was obtained by centrifugation and stored at −80°C. A Waters ACQUITY UPLC BEH Amide (2.1 mm × 50 mm, 1.7 μm, VK) was used for separation. The mobile phase consisted of Methanol (A) and acetonitrile-water (B), and the flow rate was set at 0.25 ml/min. The gradient profile was: 0–1 min (A: 90%; B: 10%), 1–8 min (A: 60%; B: 40%), 8–15 min (A: 90%; B: 10%). The sample injection volume was 2 μL. The ESI source operates in negative mode, with a capillary voltage of 2.0 kV and a desolvation temperature of 550°C. The source of the gas was set as follows: desolvation at 200 L/h and cone at 0 L/h. The collision cell pressure was 4.5 × 10^−3^ mbar. Dates were processed using MassLynx™4.1 software (Waters Corp, Milford, MA, United States). Lactulose and mannitol contents were calculated as well as the standard curve and corresponding peak area.

### Gut Microbiota Composition Analysis

To investigate the effect of ZSWF on intestinal microflora of DCI mice, feces of each group were collected by individual metabolic chamber for approximately 24 h after treatment, and 16S rDNA sequencing was performed. The metagenomic DNA from each feces sample was extracted using a QIAamp DNA Stool Mini Kit (Qiagen, Hamburg, Germany). Thereafter, the DNA concentration was determined, and the V3-V4 regions of bacterial 16S rDNA was amplified by PCR (95°C for 3 min, followed by 27 cycles at 95°C for 30 s, 55°C for 30 s, and 72°C for 45 s and a final extension at 72°C for 10 min, 10°C until halted by user) using 10 ng DNA as a template. The 16S primers 338F-ACTCCTACGGGAGGCAGCAG and 806R-GGACTACHVGGGTWTCTAAT were used as fusion primers containing Ion Torrent sequencing adapters. PCR reactions were performed in triplicate 20 μl reaction mixtures containing 4 μl of 5 × FastPfu Buffer (1 μM), 2 μl of 2.5 mM dNTPs, 0.8 μl of Forward Primer (5 μM), and 0.8 μl of Reverse Primer (5 μM), 0.4 μl of FastPfu Polymerase, 2 μl of Microbial DNA (5 ng/μl), and add ddH2O to 20 μl. Afterwards PCR products were gel-purified, and the amplicon DNA concentration was determined. Sequencing of pooled amplicons was performed with an Illumina MiSeq platform (Illumina Inc., San Diego, United States), and the resulting analysis using the MiSeq Reporter software (MSR) and the classification is based on the Greengenes database. Subsequent cord diversity analysis applied to determine *α* (within sample) and *β* (between samples) diversity. To demonstrate the clustering of different groups, the Nonmetric multidimensional scaling and weighted unifrac principal coordinate analysis (PCoA) were conducted.

### Statistical Analysis

Data were processed and analyzed using the statistical package SPSS (version 17.0), and the results were expressed as mean ± standard deviation (SD). Data were tested for normality before difference analysis, escape latency data were analyzed by repeated measures ANOVA, the remaining data were analyzed by one-way ANOVA, followed by a post hoc Tukey’s Honest Significant Difference test for multiple comparisons among the groups. A *p* value of less than 0.05 was considered to indicate statistical significance. For 16S rRNA gene sequence analysis, all reads were deposited and grouped into operational taxonomic units (OTU) at a sequence identity of 97%, and the taxonomic affiliation of the OTUs was determined with quantitative insights into microbial ecology (version 1.8.0) against the Greengenes database (version 13.5). Principal component analysis was performed using SIMCA 14.0, and metastats was used to analyze differences between groups.

## Results

### Zi Shen Wan Fang Ameliorated Cognitive Impairment and Prevented Neuron Damage in Diabetic Cognitive Impairment Mice

**FIGURE 1:**
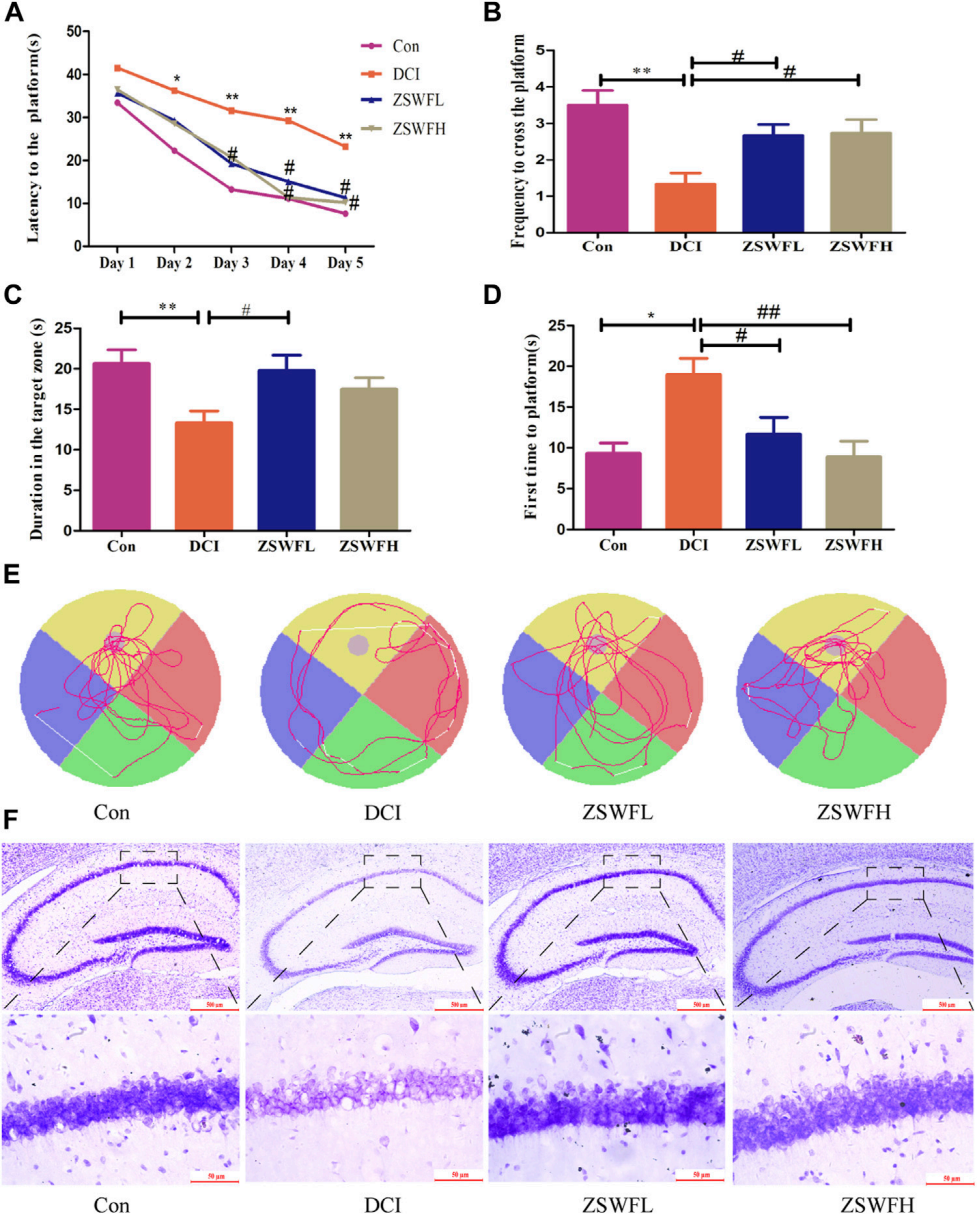
ZSWF ameliorated cognitive impairment and prevented neuron damage in DCI mice. **(A)** escape latency, **(B)** the frequency of crossing the platform, **(C)** duration in the platform quadrant, **(D)** time of first arrival at platform. These data are expressed as the mean ± SD, (*n* = 15). Escape latency data were analyzed by repeated measurement ANOVA, other data were analyzed by one-way ANOVA. **p < 0.01, *p < 0.05 vs. Con group, ^##^ *p* < 0.01, ^#^ *p* < 0.05 vs. DCI group. **(E)** representative swimming tracks of each group of mice in the space exploration experiment. **(F)** representative images of cresol violet staining in hippocampus of each group of mice.

To investigate the effect of ZSWF on the body weight of DCI mice, we analyzed the changes of body weight of each group of mice every 2 weeks during the treatment of ZSWF, and we found that ZSWF tended to increase the body weight of DCI mice, but no statistical difference (*p* > 0.05) was found, indicating that ZSWF demonstrated no effect on the body weight of DCI mice. Considering that hyperglycemia is the primary cause of cognitive dysfunction, we first investigated the effect of ZSWF on FBG in DCI mice. The results showed that no significant difference in FBG was found between the ZSWF group and the DCI group during continuous treatment (*p* > 0.05), whether the ZSWFL or ZSWFH group (Supplementary Figure S1). Next, to investigate the effect of ZSWF on cognitive impairment in DCI mice, the MWM experiment was performed. The results of the positioning navigation experiment showed that the escape latency of DCI group was significantly increased compared with the Con group (*p* < 0.05), while the escape latency of mice in the ZSWF group was significantly decreased compared with the DCI group (*p* < 0.05) (Figure 1A). Next, the results of the space exploration experiment showed that ZSWF significantly increased the frequency of crossing the platform (*p* < 0.01, *F* = 6.590) (Figure 1B) and the duration of swimming in the platform quadrant (Figure 1C), and it reduced the time for DCI mice to reach the platform for the first time (*p* < 0.05, *F* = 6.434) (Figure 1D). Moreover, the swimming trajectory of the space exploration experiment showed that the swimming trajectory of the ZSWF group is oriented, while that of the DCI group is disordered (Figure 1E).

The hippocampus is the main brain region regulating learning and memory, and the damage of hippocampus neurons is the final pathological change of cognitive dysfunction. Therefore, we investigated the protective effect of ZSWF on DCI hippocampal neurons by cresol violet staining the neuronal Nissl bodies. As described in Figure 1F, the DCI animals neuron layer is thin, irregularly and loosely arranged, intercellular spaces are widened, and the cellular structure is incomplete, even with the loss of large amounts of cells. Meanwhile, this phenomenon can be reversed by the ZSWF administration, whether ZSWFL or ZSWFH.

### Zi Shen Wan Fang Suppressed Hippocampal and Peripheral Inflammation in Diabetic Cognitive Impairment Mice

**FIGURE 2:**
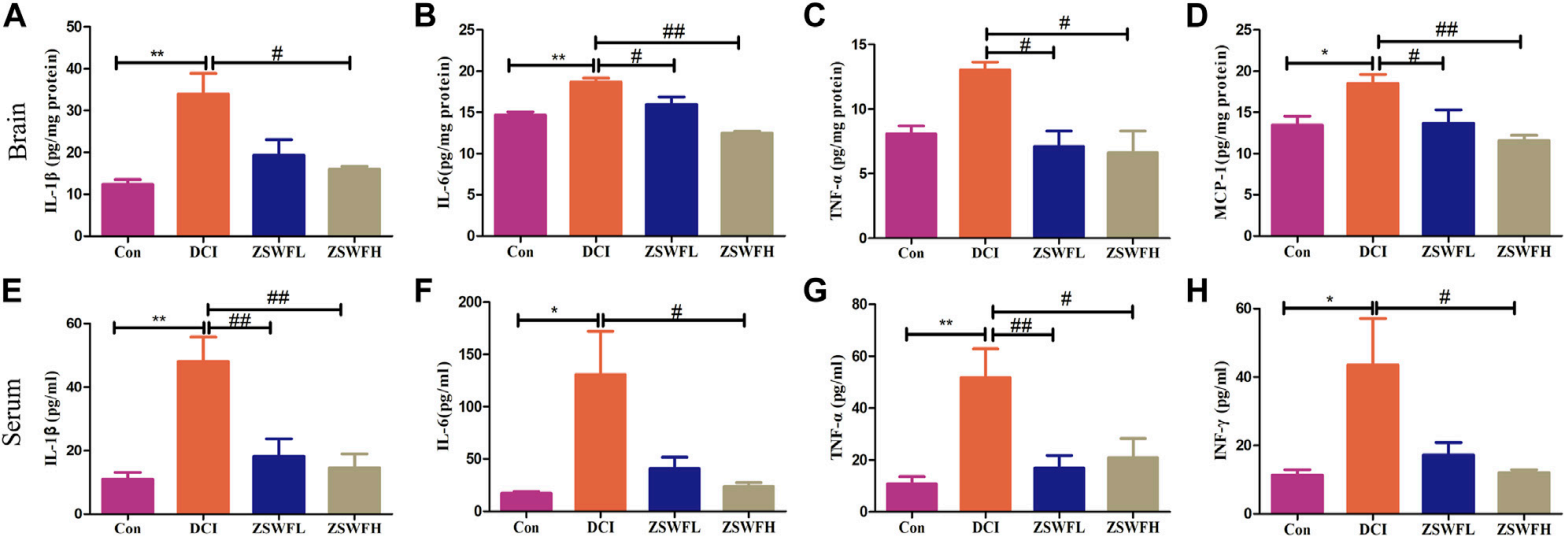
ZSWF suppressed hippocampal and peripheral inflammation in DCI mice. **(A)** IL-1β contents in the hippocampus, **(B)** IL-6 contents in the hippocampus, **(C)** TNF-α contents in the hippocampus, **(D)** MCP-1 contents in the hippocampus, **(E)** IL-1β levels in the serum, **(F)** IL-6 levels in the serum, **(G)** TNF-α levels in the serum, **(H)** INF-γ levels in the serum. All data were expressed as mean ± SD (*n* = 6). One-way ANOVA was used to compare statistical differences between groups. **p < 0.01, *p < 0.05 vs. Con group, ^##^ *p* < 0.01, ^#^ *p* < 0.05 vs. DCI group.

Cumulative studies reported that neuroinflammation is an important pathological mechanism of DCI (Rom et al., 2019), and chronic systemic inflammation is one of the important characteristics of DM (Esser et al., 2014). Thus, we measured the levels of classic proinflammatory cytokines IL-1β, IL-6, TNFα, MCP-1, and INF-γ in the hippocampus and serum. Our results indicated that the hippocampus levels of IL-1β (*F* = 8.909, *p* < 0.01) (Figure 2A), IL-6 (*F* = 20.64, *p* < 0.01) (Figure 2B), TNF-α (*F* = 6.935, *p* > 0.05) (Figure 2C), and MCP-1 (*F* = 7.033, *p* < 0.05) (Figure 2D) in DCI mice were significantly increased, and the levels of IL-1β (*F* = 10.12, *p* < 0.01) (Figure 2E), IL-6 (*F* = 4.719, *p* < 0.05) (Figure 2F), TNF-α (*F* = 6.767, *p* < 0.01) (Figure 2G), and INF-γ (*F* = 4.727, *p* < 0.05) (Figure 2H) in serum were also significantly increased. In contrast, these proinflammatory cytokines were significantly reduced in the ZSWF group compared with the DCI group (*p <* 0.05), although no significant dose dependence occurred.

### Zi Shen Wan Fang Maintains Intestinal Integrity in Diabetic Cognitive Impairment Mice

**FIGURE 3:**
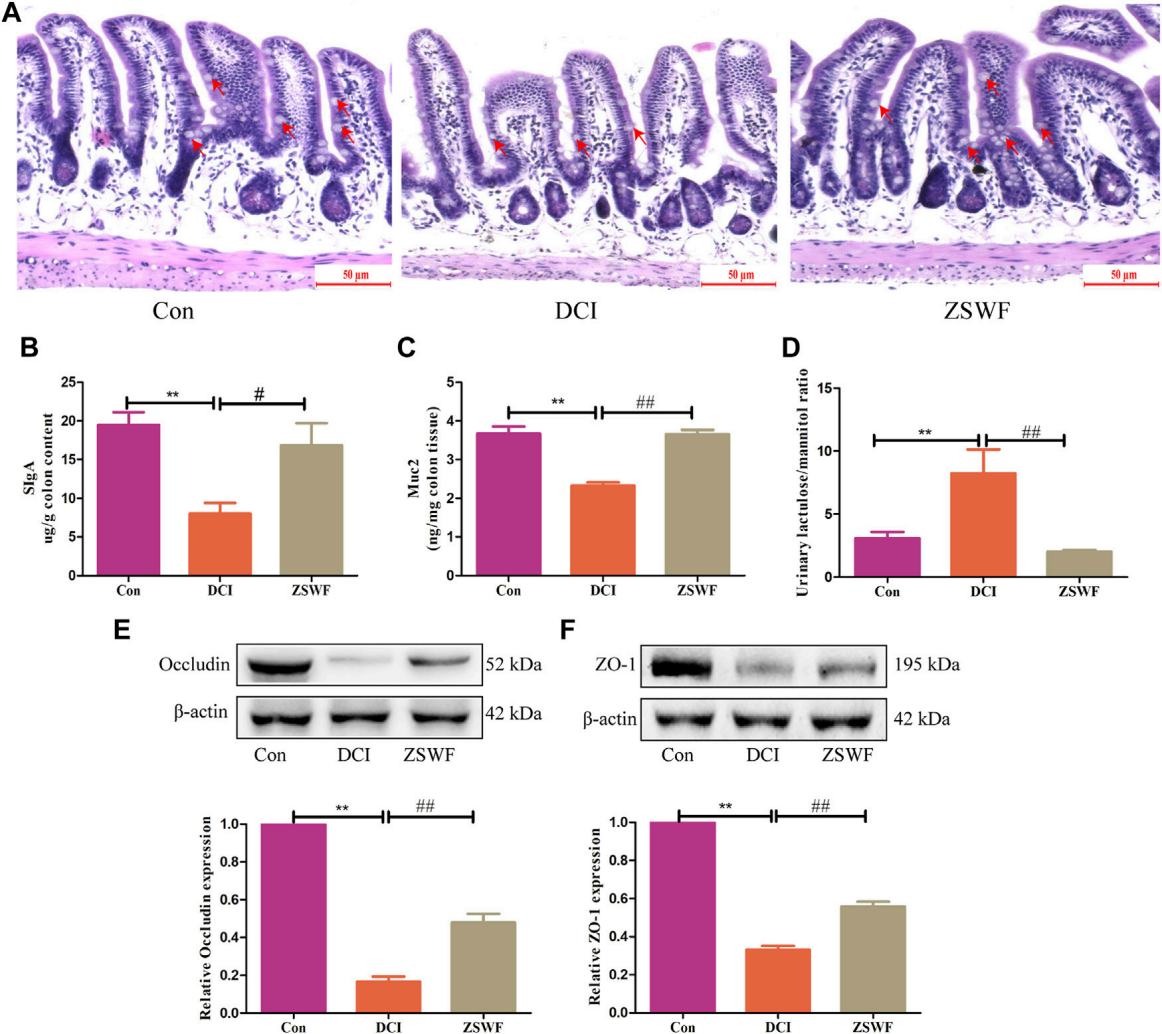
ZSWF maintains intestinal integrity in DCI mice. **(A)** representative images of colon HE staining, the goblet cells marked by the red arrow, scale bar 50 μm (*n* = 3). **(B,C)** SlgA and Muc2 contents in the colon, **(D)** lactulose to mannitol ratio, these data were expressed as mean ± SD (*n* = 5). **(E,F)** occludin and ZO-1 represented Western blotting bands and relative protein expression levels in the colon, data were expressed as mean ± SD (*n* = 3). All data were analyzed using one-way ANOVA, **p < 0.01 vs. Con group, ^##^ *p* < 0.01, ^#^ *p* < 0.05 vs. DCI group.

Given that intestinal dysbiosis in diabetes animals may affect gut integrity and subsequently lead to release of bacterial proinflammatory cytokines into the circulation, we examined the effect of ZSWF on intestinal barrier integrity in DCI mice. HE staining results showed that compared with the Con group, the colonic mucosa of mice in the DCI group was damaged, the number of goblet cells was reduced, and mucosal muscle layer was thinned, while ZSWF protected the colonic injury of DCI mice (Figure 3A). Furthermore, intestinal SIgA plays a key role in the intestinal immune system. Our data showed that compared with the Con group, the SIgA level of colon contents was significantly reduced in the DCI group (*F* = 8.092, *p* < 0.01), while ZSWF treatment significantly reversed the decreased SIgA level in DCI mice (*p* < 0.05) (Figure 3B). Next, we examined the level of Muc2 secreted by goblet cells and found that ZSWF treatment significantly increased the content of Muc2 in the DCI mice colon (*F* = 33.39, *p* < 0.01) (Figure 3C). Moreover, the urinary lactulose/mannitol (L/M) ratio was used to determine intestinal permeability and found that the urinary L/M ratio of the ZSWF group was significantly lower than that of the DCI group (*F* = 12.06, *p* < 0.01) (Figure 3D). In addition, we also investigated the effect of ZSWF on tight junction protein expression in the colon of DCI mice and found that compared with the Con group, the expression of tight junction proteins ZO-1 and occludin in the colon of DCI mice was significantly decreased, while ZSWF treatment significantly increased the expression of ZO-1(*F* = 326.8, *p* < 0.01) and occludin (*F* = 181.2, *p* < 0.01) in the colon of DCI mice (Figures 3E,F).

### Zi Shen Wan Fang Reversed Gut Microbiota Dysbiosis and Increased Short-Chain Fatty Acids in Diabetic Cognitive Impairment Mice

**FIGURE 4:**
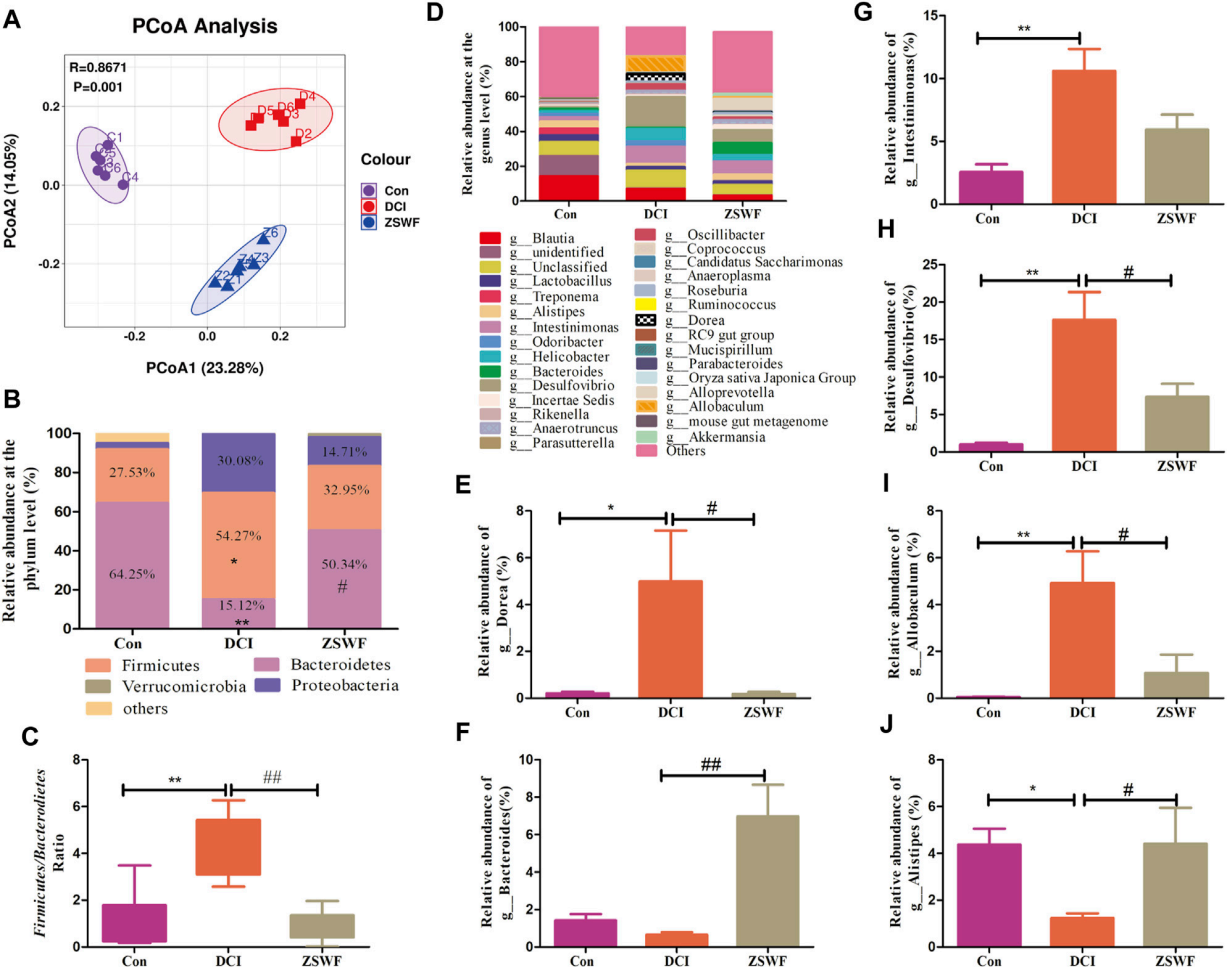
ZSWF reversed gut microbiota dysbiosis in DCI mice. **(A)** principal coordinates analysis (PcoA), **(B)** relative abundances at phylum levels, **(C)** ratio of *Firmicutes* to *Bacteroidetes,* **(D)** relative abundance of bacteria at the genus level, **(E–J)** relative abundance of *Dorea*, *Bacteroides*, *Intestinimonas*, *Desulfourbrio*, *Allobaculum*, and *Alistipes*. All data were expressed as mean ± SD (*n* = 6), and one-way ANOVA was used to compare statistical differences between groups, **p < 0.01, *p < 0.05 vs. Con group, ^##^ *p* < 0.01, ^#^ *p* < 0.05 vs. DCI group.

Gut microbiota may be an ideal target for understanding and treating DCI. To reveal the effect of ZSWF in regulating gut microbiota in DCI mice, the feces were sampled and sequenced by performing a pyrosequencing-based analysis of bacterial 16S rDNA (V3-V4 region). Weighted UniFrac distance-based PCoA revealed a distinct clustering of microbiota composition for each group (Figure 4A). At the phylum level, the DCI mice displayed an increased the relative abundance of *Firmicutes* (54.27% vs. 27.53%) and *Proteoobacteria* (30.08% vs. 3.24%) and decreased abundance of *Bacteroidetes* (15.21% vs. 64.25%) compared with Con mice. In contrast, the microbiota imbalance was ameliorated by ZSWF administration as it decreased the abundance of *Firmicutes* (32.95% vs. 54.27%) and *Proteoobacteria* (14.71% vs. 30.18%) and increased *Bacteroidetes* (50.34% vs. 15.2%) (Figure 4B). Furthermore, treatment with ZSWF reduced the ratio of *Firmicutes* to *Bacteroidetes* in DCI mice (Figure 4C). At the genus level, the relative abundance accounted for the top 30 was analyzed, and after ZSWF treatment, several important modifications of the gut microbiota composition were found. The relative abundance of *Bacteroides* (*F* = 14.48, *p* < 0.01) and *Alistipes* (*F* = 4.559, *p* < 0.05) were markedly increased, and *Dorea* (*F* = 5.396, *p* < 0.01)*, Intestinimonas* (*F* = 11.79, *p* > 0.05)*, Desuifouibrio* (*F* = 12.45, *p* < 0.05), and *Allobaculum* (*F* = 10.32, *p* < 0.05) were significantly decreased in the ZSWF administration group compared to the DCI group (Figures 4D–J). These results confirmed that the homeostasis of the gut microbiota in DCI individuals was destroyed, and ZSWF could play an important role in modulating the composition of gut microbiota on DCI mice. Furthermore, considering that short-chain fatty acids (SCFAs) are major metabolites of intestinal microbiota and play an important role in gut-brain axis communication, we examined the effect of ZSWF on fecal SCFAs concentration and found that ZSWF could increase the concentration of SCFAs in feces of DCI mice (Supplementary Figure S3).

### Microbiota Ablation With Antibiotics Eliminated Zi Shen Wan Fang in Improving Cognitive Function and Reducing Neuroinflammation as Well as Systemic Inflammation of Diabetic Cognitive Impairment Mice

**FIGURE 5:**
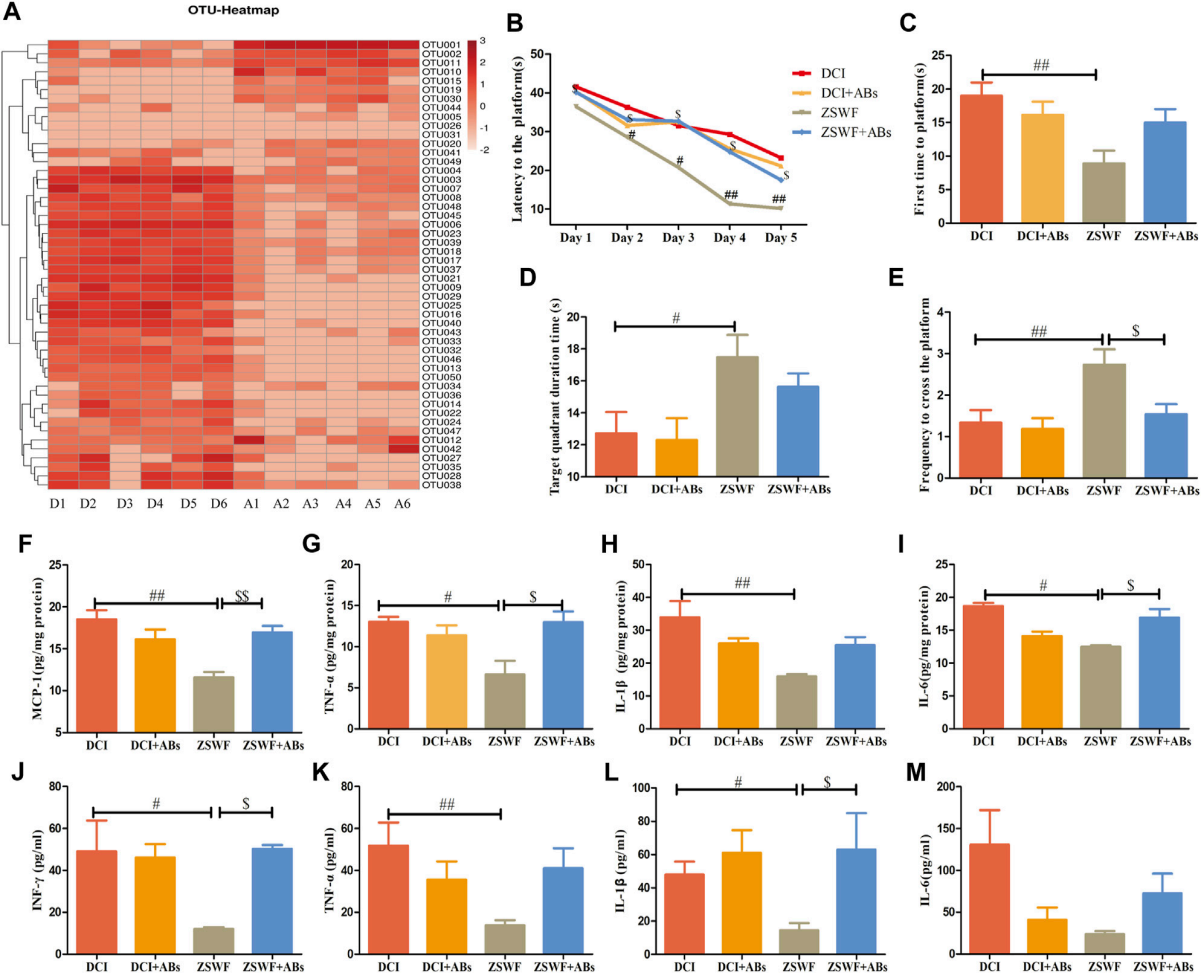
Microbiota ablation with antibiotics eliminated the effects of ZSWF on improving cognitive function and reducing inflammation in DCI mice. **(A)** effects of antibiotic treatment on relative abundance of intestinal bacteria at genus level in DCI mice. The heat map represents the relative sequence abundance of Top50 OTU of genus level bacteria. The redder color represents more bacteria composition or the higher relative abundance of genus level bacteria. The horizontal axis represents groups, D1–D6 are six samples from the DCI group, and A1–A6 are six samples from the antibiotic group. **(B)** escape latency, **(C)** time of first arrival at platform, **(D)** duration in platform quadrant, **(E)** the frequency of crossing the platform. These data were expressed as mean ± SD (*n* = 15). Escape latency data were analyzed by repeated measurement ANOVA, and other data were analyzed by one-way, ^##^ *p* < 0.01, ^#^ *p* < 0.05 vs. DCI group, ^$^ *p* < 0.05 vs. ZSWF group. **(F)** MCP-1 contents in the hippocampus, **(G)** TNF-α contents in the hippocampus, **(H)** IL-1β contents in the hippocampus, **(I)** IL-6 contents in the hippocampus, **(J)** INF-γ levels in the serum, **(K)** TNF-α levels in the serum, **(L)** IL-1β levels in the serum, **(M)** IL-6 levels in the serum. These data were expressed as mean ± SD (*n* = 6), one-way ANOVA was used to compare statistical differences between groups, ^##^ *p* < 0.01, ^#^ *p* < 0.05 vs. DCI group, ^$$^ *p* < 0.01, ^$^ *p* < 0.05 vs. ZSWF group.

The above results suggest that the gut microbiota-brain axis plays an important role in ZSWF in improving cognition impairment of DCI mice. To further investigate the relationship between the improvement of cognitive function and the regulation of intestinal microbiota by ZSWF, a cocktail of oral antibiotics was used to eliminate the gut microbiota. The mice were administrated with antibiotics in the drinking water starting 7 days before ZSWF treatment and throughout the experiment. First, we found that antibiotics demonstrated no effect on FBG in DCI mice (Supplementary Figure S4). Furthermore, antibiotic treatment significantly reduced the amount of OTU in the feces of DCI mice (Figure 5A), suggesting that most intestinal microbiota were eliminated. Antibiotics alone did not significantly alter cognitive function and inflammation in the DCI group, suggesting antibiotics did not further impair cognitive function and increase inflammation induced diabetes (Figures 5B–E). Of note, the antibiotic treatment abrogated the beneficial effects of ZSWF treatment in DCI mice. We found that the cognition improvement with ZSWF was abolished by antibiotics treatment, with longer escape times (Figure 5B), longer times of first to arrival platform (Figure 5C), lower times in the target quadrant (Figure 5D), and lower frequency to cross platform (Figure 5E). Moreover, we also observed that the effect of ZSWF on reducing hippocampus and serum proinflammatory cytokines was eliminated by antibiotic intervention (Figures 5F–M). Meanwhile, the effect of ZSWF intervention on increasing fecal SCFAs in DCI mice was eliminated (Supplementary Figure S5). These results suggest that maintaining intestinal microflora homeostasis is a critical pathway for ZSWF to reverse cognitive function and reduce inflammation in DCI mice.

## Discussion

Cognitive dysfunction is one of the complications of diabetes, and no clinically approved treatment drug exists. Previous cumulative studies showed that DCI is closely associated with intestinal microbiome dysregulation (Xu et al., 2017; Zhang et al., 2021a), suggesting that maintaining intestinal microbiome homeostasis may be an effective strategy to prevent and treat DCI. In this study, we investigated the effects of ZSWF on the cognitive function of DCI mice, and we explored the mechanism from the perspective of gut-microbial-brain axis. Next, we present evidence that suggests that ZSWF supplementation ameliorated the diabetes-induced cognitive dysfunction and reduced neuroinflammation and systemic inflammation. Furthermore, ZSWF treatment protected intestinal barrier integrity and maintained intestinal microbiota homeostasis in DCI mice. In particular, the effects of ZSWF treatment on improving diabetes-induced cognitive impairment and inflammation were eliminated after antibiotic treatment deleted intestinal bacteria, highlighting the essential role of gut microbiota in improving cognitive function of ZSWF.

In this study, we established a diabetic mouse model according to the method previously reported (Kusakabe et al., 2009). Given that our previous studies confirmed a certain incidence of diabetes-induced cognitive impairment (Song et al., 2017), mice with cognitive impairment were screened by MWM after 8 weeks of hyperglycemic stimulation. Our screening results revealed that diabetic mice demonstrated an approximately 80% chance of developing cognitive impairment (Unpublished). Then, we treated DCI mice with ZSWF extract for 8 weeks. FBG was measured weekly during treatment, and cognitive function was assessed after treatment. Likewise, we found that ZSWF significantly improved cognitive function and protected hippocampal neuron damage in DCI mice. However, this study found that ZSWF could not improve FBG, the initial pathological factor of DCI mice, suggesting that improving the subsequent complex pathological links mediated by hyperglycemia may be the mechanism by which ZSWF improves DCI.

Accumulated evidence suggests that neuroinflammation is an important pathological mechanism of DCI (Muriach et al., 2014; Jeong et al., 2021). IL-6, IL-1β, and TNF-α are common proinflammatory cytokines, which are significantly altered in a variety of acute and chronic inflammatory diseases. IFN-γ is secreted mainly by natural killer cells and natural killer T cells and plays a role in innate immunity. A large number of excellent previous studies confirmed that the diabetic hyperglycemic environment mediates changes in innate immune system function and significantly increases IFN-γ levels. MCP-1 is a major chemokine that recruits monocyte/macrophage to the site of tissue injury and plays a critical role in microvascular complications of diabetes. Therefore, in order to investigate the effects of ZSWF on the system and neuroinflammation of DCI mice, we used the ELISA kit to detect the changes of the above cytokines, and we found that the levels of these proinflammatory factors in the hippocampus of DCI mice were significantly increased, which was consistent with previous reports (Wang et al., 2019; Zeinivand et al., 2020). Likewise, our current study also revealed that ZSWF reduced hippocampal proinflammatory cytokines levels in DCI mice. Also, we found that ZSWF inhibited the activation of brain-resident immune cells (microglia and astrocytes) in DCI mice (unpublished). In addition to brain-resident immune cells releasing proinflammatory cytokines, peripheral circulating proinflammatory cytokines crossing the blood-brain barrier is also a key pathologic pathway of neuroinflammation. In a similar manner, our results showed that ZSWF significantly reduced peripheral circulating proinflammatory cytokines in DCI mice. These results suggest that the reduction of peripheral circulation and neuroinflammation is the mechanism of ZSWF to improve DCI. However, studies showed that the bioavailability of main chemical components of Anemarrhenae Rhizoma and Phellodendri Chinensis Cortex is low (Singh and Chaudhuri, 2018; Baldim et al., 2020), and our previous *in vivo* chemical analysis of ZSWF also showed that most components in Cistanches Herba were concentrated in feces (Zheng et al., 2018), suggesting that regulation of gastrointestinal function or intestinal flora may play a crucial role in the reversal of DCI inflammation by ZSWF.

Increasing evidence also indicates that diabetes-induced systemic inflammation is mainly caused by impaired intestinal barrier integrity and dysbiosis of intestinal flora (Cani et al., 2008; Cani et al., 2009; Li et al., 2019). Our detection results of intestinal barrier integrity related parameters showed that the intestinal barrier integrity was damaged in DCI mice, which was consistent with previous reports (Visser et al., 2010; Li et al., 2022; Xiao et al., 2022). Likewise, our results show that ZSWF exhibits a protective effect on intestinal barrier integrity in DCI mice by increasing colon tight junction protein expression (ZO-1 and occludin) and mucin (Muc2) content, as well as colon content immunoglobulin A (SlgA) levels. These results systematically confirmed that ZSWF maintained intestinal barrier integrity in DCI mice.

Intestinal microbiome is a key component of the intestinal barrier, and its homeostasis can lead to an increase in the permeability of the intestinal barrier. The results of our 16S rDNA sequencing revealed that the intestinal microbiome composition of DCI mice was significantly altered, and ZSWF demonstrated a significant reversal effect on the microbiome disorder of DCI mice. The relative abundance results of phylum level bacteria showed that DCI mice increased the relative abundance of *Firmicutes* and *Proteoobacteria*, and they significantly decreased the abundance of *Bacteroidetes*, which was consistent with previous reports (Zhu et al., 2016). More notably, ZSWF improved the imbalance of intestinal bacteria, as demonstrated by a decreased ratio of *Firmicutes* to *Bacteroides*. At the genus levels, our results showed that ZSWF treatment significantly increased the abundance of *Bacteroides* and *Alistipes*, and it decreased the abundance of *Desulfouibrio*, *Dorea*, and *Allobaculum*. Clinical studies linked changes in *Bacteroides* to cognitive and neurodegenerative diseases (Cattaneo et al., 2017; Saji et al., 2019). Also, previous studies found that *Bacteroides* can reduce inflammation (Tan et al., 2019) and protect intestinal mucosal permeability (Hooper et al., 2001). Studies found that the abundance of *Alistipes* is significantly reduced in patients with mild cognitive impairment, suggesting that *Alistipes* is negatively correlated with cognitive function (Zhang et al., 2021b). *Desulfovibrio* is a proinflammatory bacterium (Simpson et al., 2021) and can produce hydrogen sulfide, which demonstrates a cytotoxic effect (Gibson, 1990). The above evidence suggested that ZSWF could maintain intestinal microbiome homeostasis in DCI mice, which was reflected in that ZSWF increased the relative abundance of beneficial bacteria in DCI mice and decreased the abundance of bacteria that produced cytotoxic substances. Antibiotic cocktail treatment is an appropriate method to explore the effects of intestinal bacteria on physiology and disease in animal (Kennedy et al., 2018). Data from ZSWF combined with antibiotic therapy revealed that oral antibiotics did not improve or worsen FBG, cognitive function, as well as inflammation in DCI mice. However, oral antibiotics treatment eliminated the effect of ZSWF on cognitive function and inflammation in DCI mice, suggesting that gut microbes are necessary to ZSWF to improve cognitive function and inflammation in DCI mice.

The regulation of intestinal flora by ZSWF in DCI mice may be bidirectional. On the one hand, the chemical composition of ZSWF maintained the composition of intestinal microbes in DCI mice, and on the other hand, intestinal microbes in DCI mice metabolized the components with low bioavailability of ZSWF. The potential active components of ZSWF regulating intestinal flora of DCI mice may be polysaccharides in Cistanches Herba and alkaloids in Phellodendri Chinensis Cortex. Previous studies confirmed that Cistanche polysaccharides demonstrate a clear regulatory effect on intestinal flora (Fu et al., 2020; Fan et al., 2021; Gao et al., 2021). Although accumulative studies confirmed that berberine, the main alkaloid component of Phellodendri Chinensis Cortex, can protect cognitive function (Aski et al., 2018; Shinjyo et al., 2020; Yi et al., 2021), subsequent studies gradually recognized that regulating intestinal flora may be the initial link of berberine improving cognitive function (Zhang et al., 2020b; Habtemariam, 2020). The ZSWF components metabolized by intestinal flora of DCI mice may be saponins in Anemarrhenae Rhizoma (Tian et al., 2016; Dong et al., 2021). Furthermore, besides the immune signaling pathway investigated in this study, the mechanism of ZSWF regulating intestinal flora to improve DCI may also include the following aspects: first, regulating the tryptophan-kynurenine metabolic pathway. Previous cumulative studies confirmed that gut microbiota plays an important role in regulating tryptophan-kynurenine metabolism (Kennedy et al., 2017; Agus et al., 2018; Deng et al., 2021), and several studies also found that tryptophan-kynurenine metabolism is involved in the regulation of glutamate neurotransmitters and synaptic excitability, which is crucial for the protection of cognitive function (Oxenkrug, 2007; Forrest et al., 2015; Tanaka et al., 2020; Bakker et al., 2021). More notably, our previous study found abnormal kynurenine metabolism in DCI mice, and ZSWF treatment could improve kynurenine metabolism in DCI mice (Yin et al., 2022). These studies suggest that regulation of tryptophan-kynurenine metabolic pathway may be the mechanism by which ZSWF improves cognitive function by maintaining intestinal microbiome homeostasis in DCI mice. Second, the increase the level of SCFAs derived from gut microbiota. As the main metabolite of gut microbiota, SCFAs have been reported to ameliorate cognitive impairment mediated by a variety of etiologies, including DCI (Lee et al., 2020; Enry et al., 2021; Qian et al., 2021; Zheng et al., 2021). Our previous study also found that ZSWF increased fecal SCFAs levels in DCI mice (unpublished). These results suggest that increasing the level of SCFAs derived from gut microbiota may also be the mechanism by which ZSWF regulates intestinal microflora to improve cognitive function in DCI mice. However, this study still demonstrates some limitations. For example, although our previous studies identified the main chemical components of ZSWF extract *in vivo* and *in vitro*, including the main archetypes and metabolic components in feces (Zheng et al., 2018), which components are involved in maintaining intestinal microbiota homeostasis and their mechanisms remain unclear. Moreover, since no drug with a definite therapeutic effect for DCI exists in clinical practice at present, this study did not select a suitable positive control.

## Conclusion

ZSWF can improve cognitive dysfunction and neuroinflammation in DCI mice, while oral antibiotics can partially eliminate these effects, suggesting that maintaining intestinal microbial homeostasis may be the underlying mechanism of ZSWF to improve cognitive function in DCI mice.

## Data Availability

The datasets presented in this study can be found in online repositories. The names of the repository/repositories and accession number(s) can be found below: https://www.ncbi.nlm.nih.gov/bioproject/PRJNA836714/.

## Abbreviations

ZSWF, Zi Shen Wan Fang; DCI, diabetic cognitive impairment; STZ, streptozotocin; ABs, antibiotic; MWM, morris water maze; HE, hematoxylin-eosin; UPLC-MS/MS, high performance liquid chromatography tandem mass spectrometry; DM, diabetes mellitus; SCFAs, short-chain fatty acids; HFD, high-fat diet; Muc2, Mucin-2; SIgA, secretory immunoglobulin A; PCoA, weighted unifrac principal coordinate analysis; FBG, fasting bood glucose.
