# Supplementary material for: Zi Shen Wan Fang Attenuates Neuroinflammation and Cognitive Function Via Remodeling the Gut Microbiota in Diabetes-Induced Cognitive Impairment Mice
Source: Front Pharmacol. 2022 Jul 15;13:898360. doi: 10.3389/fphar.2022.898360 (PMC9335489; doi:10.3389/fphar.2022.898360)
Supplement: Supplementary file 1 [file Table1.docx]

Supplementary Information for

**Zi Shen Wan Fang attenuates neuroinflammation and cognitive function via remodeling the gut microbiota in diabetes-induced congitive impairment mice**

Jiangwei Shi^1, #^, Qingsheng Yin^2, #^, Lin Zhang^2^, Mengqing Guo^2^, Huhu Li^3^, Liuyi Yuan^2^, Zixuan Wang^2^, Pengwei Zhuang ^2, 4,5,*^, Yanjun Zhang^4,5, *^

^1^ Department of Integrated Rehabilitation, First Teaching Hospital of Tianjin University of Traditional Chinese Medicine, Tianjin, China

^2^ Chinese Materia Medica College, Tianjin University of Traditional Chinese Medicine, Tianjin, 301617, China

^3^ School of Integrative Medicine, Tianjin University of Traditional Chinese Medicine, Tianjin 301617,China

^4^ State Key Laboratory of Component-based Chinese Medicine, Tianjin University of Traditional Chinese Medicine, Tianjin, 301617, China

^5^ Haihe Laboratory of Modern Chinese Medicine, Tianjin University of Traditional Chinese Medicine, Tianjin, 301617, China

^*^Correspondence to:

Yanjun Zhang and Pengwei Zhuang, Tianjin University of Traditional Chinese Medicine, Jinghai District, Tianjin, 301617, China. Tel: +86-22-59596138. E-mail: [zyjsunye@163.com](mailto:zyjsunye@163.com), zhuangpengwei@163.com.

^#^ These authors contributed equally.

**The supplementary materials for this manuscript include the following:**

Figure S1-S6.

**1 Method**

**1.1 Fecal Short-chain Fatty Acids Analysis**

The fecal was weighted and EBA was added as an internal standard, the sample was fully homogenized with 1.0 ml of 0.5 M oxalic acid for 5 min and centrifuged for 10 min. The supernatant was filtered with a 0.22 um nylon membrane and was transferred to a gas chromatograph (GC) that instrument equipped with a flame ionization detector (FID). Concentrations of individual SCFA were measured on a fused-silica capillary column with a free fatty acid phase (DB-FFAP) and dimensions of 30 m×250 μm×0.25 μm. The high purity nitrogen (more than 99.99%) was used as the carrier gas and the initial flow velocity is 0.8 mL/min. The high purity hydrogen (more than 99.99%) was used as the auxiliary gas. The initial oven temperature was 60 ℃ and gradually raised to 220 ℃ at 20 ℃/min, held for 1.0 min, and then increased to 250 ℃ at 20 ℃/min. The sample (1μl) was injected with split mode that ratio of 50 to 1. The FID temperature and injection port was 280 and 250 ℃, respectively. The SCFAs were quantified using the internal standard curve method.

**1.2 Statistical Analysis**

Data was processed and analyzed using the statistical package SPSS (version 17.0), and the results were expressed as means ± standard deviation (SD). Data was tested for normality before difference analysis and analyzed by one-way ANOVA, followed by a post hoc Tukey’s Honest Significant Difference test for multiple comparisons among the groups, a p value of less than 0.05 was considered to indicate statistical signifcance.

**2 Results**

**2.1 Effects of ZSWF on body weight of DCI mice**

To investigate the changes of body weight in mice during the progression of DCI and the improvement effect of ZSWF on body weight in DCI mice, the body weight of each group was measured every two weeks during ZSWF treatment. The results showed that compared with the Con group, the body weight of mice in the DCI group was significantly decreased (*p*<0.05), but there was no more severe weight loss in the course of DCI disease. Unfortunately, ZSWF treatment tended to increase the body weight of DCI mice, but there was no statistical difference (*p*>0.05), suggesting that ZSWF did not improve the body weight of DCI mice (Fig. S1).

**2.2 Effects of ZSWF on FBG in DCI mice**

Considering that hyperglycemia is the primary cause of cognitive dysfunction, we first investigated the effect of ZSWF on FBG in DCI mice. The results showed that compared with Con group, FBG in DCI group was significantly increased (*p*<0.01), and FBG did not fluctuate significantly during DCI progression. Unfortunately, there was no effect on FBG in DCI mice during continuous treatment with ZSWF, either in the ZSWFL or ZSWFH group (*p*>0.05) (Fig. S2), suggesting that ZSWF did not improve the initial etiology of DCI.

**2.3 Effect of ZSWF on SCFAs in feces of DCI mice**

Considering that SCFAs are major metabolites of intestinal microbiota and play an important role in gut-brain axis communication, we examined the effect of ZSWF on fecal SCFAs concentration. Notably, the DCI group mice markedly decreased the concentration of acetate (F=22.02, *p*<0.01) (Fig. S3A), propionate (F=12.20, *p*<0.01) (Fig. S3B) and butyrate (F=7.174, *p*<0.01) (Fig. S3C) in the fecel. On the contrary, the sharp decline of SCFAs concentration was significantly ameliorated by ZSWF administration (*p*<0.05), especially acetate and propionate. These results showed that ZSWF could increase the concentration of SCFAs in facel of DCI mice.

**2.4 Effects of antibiotic intervention on FBG in DCI mice**

To investigate whether deletion of some intestinal microorganisms by antibiotics would aggravate or improve DCI mice, FBG of each group during antibiotic intervention was measured. Our results showed that antibiotic treatment had no effect on FBG in DCI mice, and antibiotic combined with ZSWF treatment had no effect on FBG in DCI mice (*p*>0.05) (Fig. S4), indicating that deletion of intestinal bacteria did not affect FBG in DCI mice.

**2.5 Microbiota ablation with antibiotics eliminated the effect of ZSWF on increasing SCFAs content in feces of DCI mice**

To investigate the effect of ZSWF on SCFAs after microbial ablation, SCFAs content in feces of mice in each group was detected. The results showed that antibiotics ablated microorganisms had no significant effect on the content of SCFAs in DCI mice (*p*>0.05). Interestingly, antibiotic ablative microorganisms counteracted the effect of ZSWF on increasing SCFAs content in DCI mice, especially the effects of ZSWF on increasing acetic acid (F=5.736, *p*<0.01) and propionic acid (F=9.981, *p*<0.05) in the feces of DCI mice (Fig. S5).

**Figure**

**Figure S1. Effects of ZSWF on body weight of DCI mice.**

**
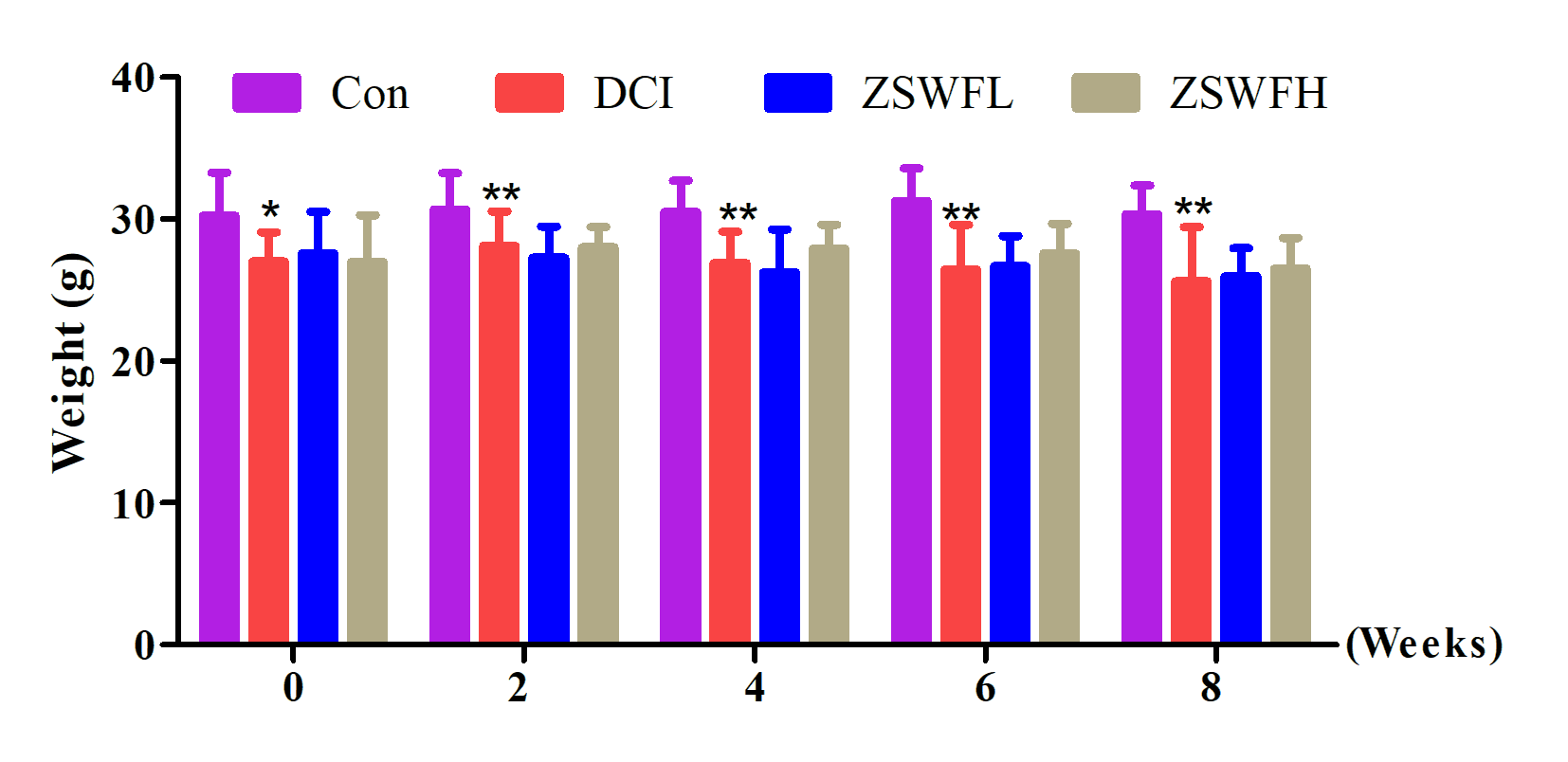
**

**Figure S2. Effects of ZSWF on FBG in DCI mice.**


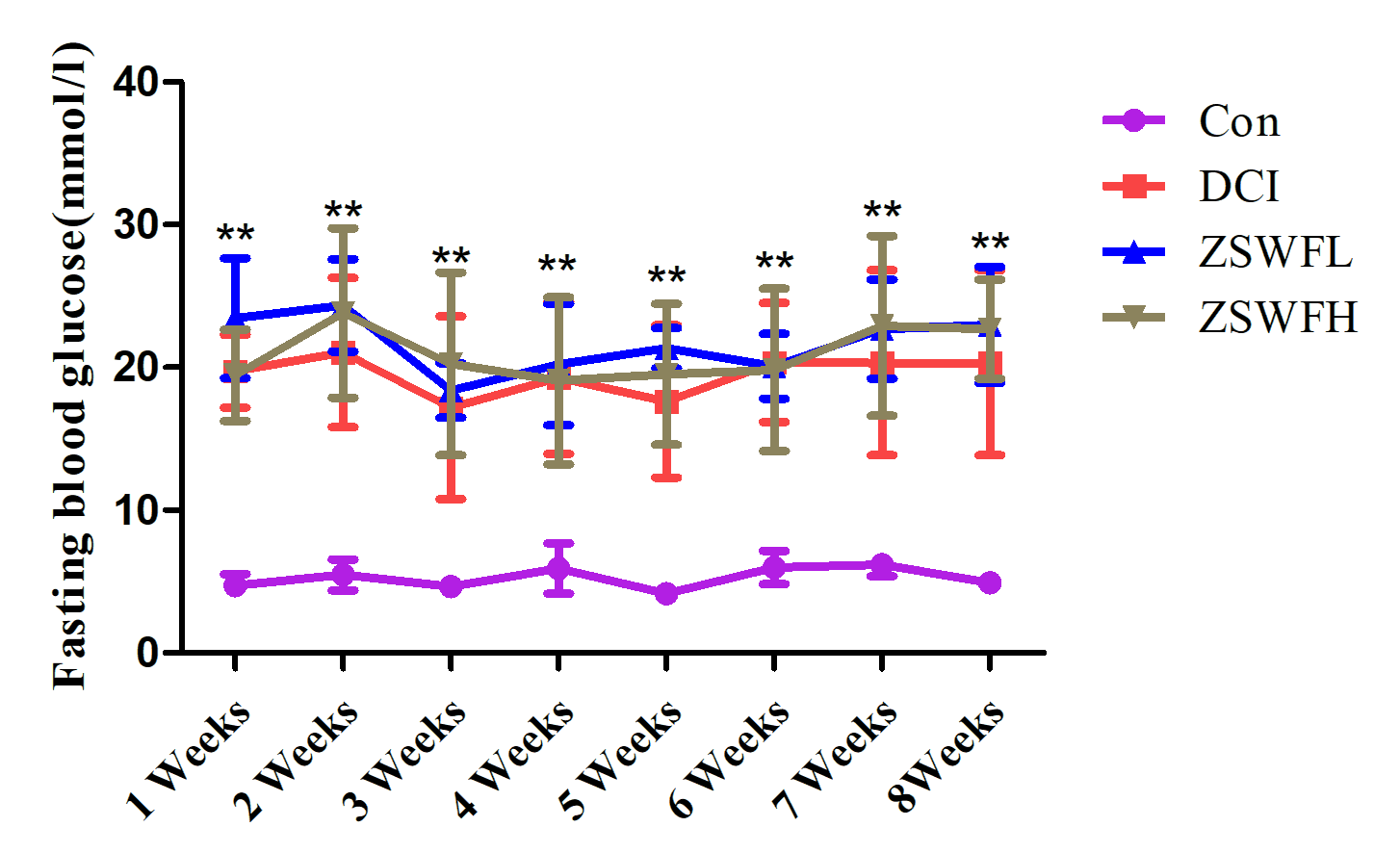


**Figure S3. Effect of ZSWF on SCFAs in feces of DCI mice**.


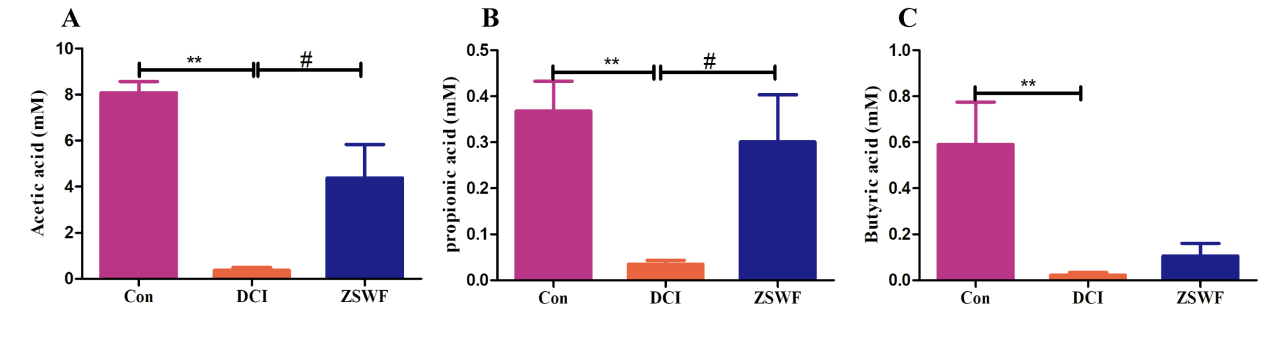


**Figure S4. Effects of antibiotic intervention on FBG in DCI mice.**

**
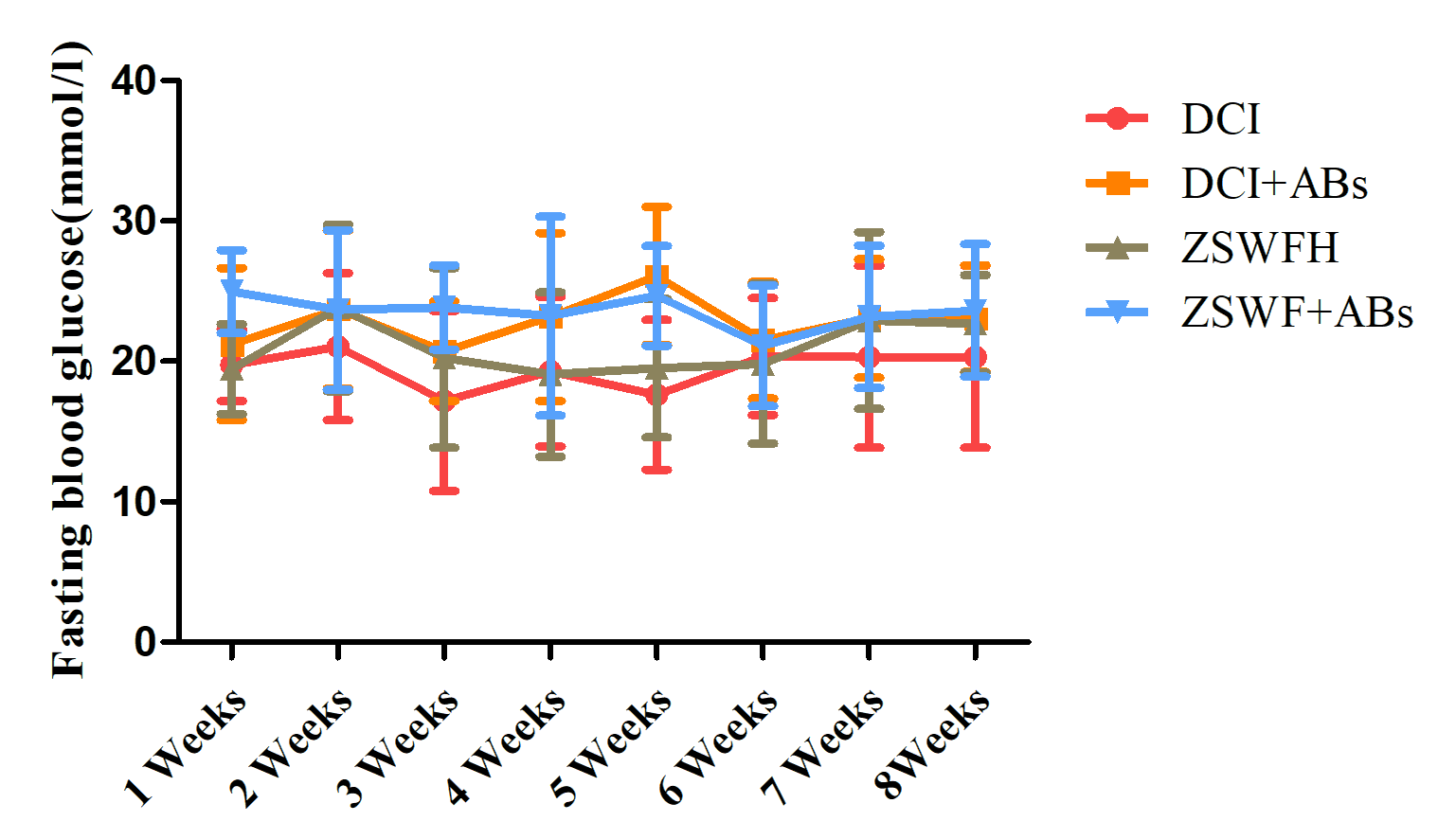
**

**Figure S5. Microbiota ablation with antibiotics eliminated the effect of ZSWF on increasing SCFAs content in feces of DCI mice.**

**
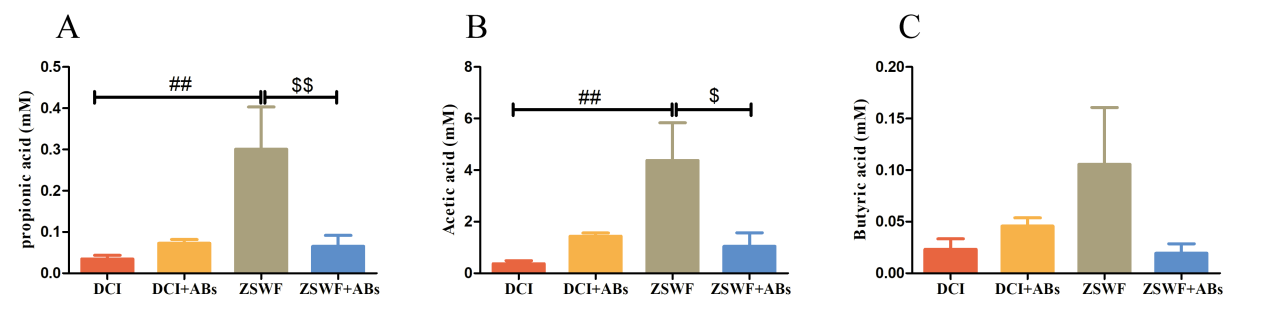
**

**Figure S6. Zi Shen Wan Fang attenuates neuroinflammation and cognitive function via remodeling the gut microbiota in diabetes-induced cognitive impairment mice.**

**
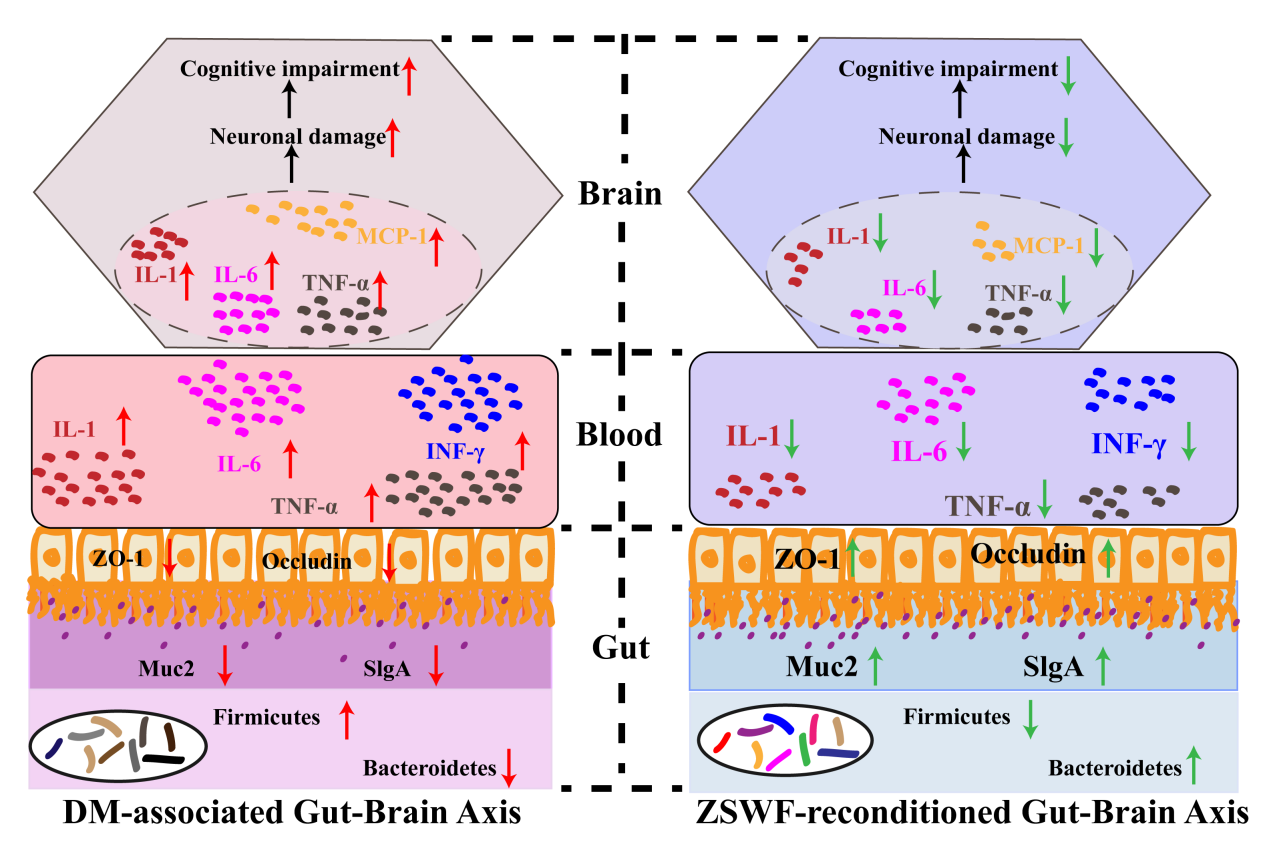
**

**Figure legends**

Figure S1. Effects of ZSWF on body weight of DCI mice. Data was expressed as the means ± SD (n=15), ^**^*p*<0.01, ^*^*p*<0.05 *vs*. Con group.

Figure S2. Effects of ZSWF on FBG in DCI mice. Data was expressed as the means ± SD (n=15), ^**^*p*<0.01 *vs*. Con group.

Figure S3. Effect of ZSWF on SCFAs in feces of DCI mice. (A-C) Acetic acid, propionic acid, butyric acid concentration in feces. Data was expressed as meas±SD (n=6), ^**^*p*<0.01 *vs*. Con group, ^#^*p*<0.05 *vs.* DCI group.

Figure S4. Effects of antibiotic intervention on FBG in DCI mice. Data was expressed as the means ± SD (n=15).

Figure S5. Microbiota ablation with antibiotics eliminated the effect of ZSWF on increasing SCFAs content in feces of DCI mice. (A-C) Acetic acid, propionic acid, butyric acid concentration in feces. Data was expressed as meas±SD (n=6), ^##^*p*<0.01 *vs.* DCI group, ^$$^*p*<0.01, ^$^*p*<0.05 *vs.* ZSWF group.

Figure S6. Schematic diagram of the interplay between the microbiota and the gut-brain axis in DCI and ZSWF intervention.
